# Dental Pain and Worsened Socioeconomic Conditions Due to the COVID-19 Pandemic

**DOI:** 10.1177/00220345211005782

**Published:** 2021-04-01

**Authors:** Y. Matsuyama, J. Aida, K. Takeuchi, S. Koyama, T. Tabuchi

**Affiliations:** 1 Department of Global Health Promotion, Tokyo Medical and Dental University, Tokyo, Japan; 2 Department of Oral Health Promotion, Graduate School of Medical and Dental Sciences, Tokyo Medical and Dental University, Tokyo, Japan; 3 Division for Regional Community Development, Liaison Center for Innovative Dentistry, Graduate School of Dentistry, Tohoku University, Sendai, Japan; 4 Department of Preventive Medicine, Nagoya University Graduate School of Medicine, Nagoya, Japan; 5 Cancer Control Center, Osaka International Cancer Institute, Osaka, Japan

**Keywords:** public health, socioeconomic factors, dental caries, periodontal diseases, health services accessibility, psychological distress

## Abstract

The coronavirus disease 2019 (COVID-19) pandemic has led to economic contraction and significant restrictions on society. The shock to the economy could lead to a deterioration of physical health outcomes, including dental health. The present study investigated the association between worsened socioeconomic conditions due to the COVID-19 pandemic and dental pain in Japan. The mediating effects of psychological distress and oral health–related behaviors were also evaluated. Cross-sectional data from the Japan COVID-19 and Society Internet Survey conducted from August to September 2020 (*n* = 25,482; age range, 15–79 y) were analyzed. Multivariable logistic regression models were fitted to evaluate the independent associations of household income reduction, work reduction, and job loss due to the COVID-19 pandemic with dental pain within a month. Dental pain was reported by 9.8%. Household income reduction, work reduction, and job loss were independently associated with dental pain after adjusting for confounders (odds ratios: 1.42 [95% confidence interval (CI), 1.28−1.57], 1.58 [95% CI, 1.41−1.76], 2.17 [95% CI, 1.64−2.88], respectively). The association related to household income reduction was mediated by psychological distress, postponing dental visits, toothbrushing behavior, and between-meals eating behavior by 21.3% (95% CI, 14.0−31.6), 12.4% (95% CI, 7.2−19.6), 1.5% (95% CI, −0.01 to 4.5), and 9.3% (95% CI, 5.4−15.2), respectively. Our findings showed that worsened socioeconomic conditions due to the COVID-19 pandemic deteriorated dental health. Policies that protect income and job loss may reduce dental health problems after the pandemic.

## Background

The coronavirus disease 2019 (COVID-19) has had a devastating impact on society. Most countries have implemented policy measures to minimize person-to-person transmission, including isolation, lockdowns, and widespread closures, which have contributed to controlling the infection but posed severe economic contraction (Ozili and Arun 2020). The details of the indirect shock of the pandemic are under investigation; however, it has been estimated that 17.3% of total working hours were lost in the second quarter of 2020, which led to a 10.7% decline in labor income globally (International Labour Organization 2020). In the United States, 25% of adults had a household member who had been laid off work, and 46% of adults with lower income had problems paying their bills as a result of the pandemic (Pew Research Center 2020). In Japan, the unemployment rate was 3.1% in October 2020, which is not high compared to other countries but has been rising since the pandemic onset (Ministry of Internal Communications and Affairs 2020).

A reduction in socioeconomic conditions due to crisis is detrimental to physical and mental health (Riumallo-Herl et al. 2014; Niedzwiedz et al. 2017; Mazeikaite et al. 2019). During the COVID-19 pandemic, many health outcomes and health-related behaviors, such as mental health (Bäuerle et al. 2020; Ettman et al. 2020), obesity (Jia et al. 2021), physical activity (Jia et al. 2021), and smoking (Rossinot et al. 2020), have become worse. Vulnerable populations are affected more severely, and health inequalities have been widened by the pandemic (Kawachi 2020).

Dental pain is a significant health problem that reduces quality of life and well-being (Santos et al. 2019). Lower socioeconomic conditions, stressful circumstances, less dental care utilization, and unfavorable oral health–related behaviors have been reported as risk factors for dental pain (Kuhnen et al. 2009; Medina-Solís et al. 2017; Tsuchiya et al. 2019). Under the COVID-19 pandemic, higher anxiety and depression are reported among those who lost their income (Hertz-Palmor et al. 2020) and those who had insecure jobs (Ganson et al. 2021). People in worsened socioeconomic circumstances may be unable to afford costs associated with good oral health such as dental fees. Previous studies have shown that scarcity of resources limits dental services utilization (Babazono et al. 2008; Listl 2012). Thus, dental pain might increase with the negative economic impact of the COVID-19 pandemic. The present study aimed to 1) investigate the association of worsened socioeconomic conditions (i.e., household income reduction, work reduction, and job loss) due to the COVID-19 pandemic with dental pain and 2) evaluate the mediating effect of psychological distress and oral health–related behaviors on the association related to household income reduction.

## Methods

### Study Participants and Setting

A cross-sectional study was conducted by analyzing data from the Japan “COVID-19 and Society” Internet Survey (JACSIS) study. The ethics committee at Osaka International Cancer Institute approved the study protocol (approval number: 20084). From August 25, 2020, to September 30, 2020, questionnaires were distributed to 224,389 candidates, who were selected from the panelists at a Japanese Internet research company (Rakuten Insight, Inc.) to represent the Japanese population regarding age, sex, and residential prefecture by using a simple random sampling procedure. All participants provided web-based informed consent at registration. The survey continued until the number of respondents reached the targeted sample size (*n* = 28,000). We excluded 2,518 respondents who had provided invalid responses to the questionnaire (i.e., those who we speculated had not read the questions). We defined the invalid responses as follows: respondents who, when asked to select the second item from the bottom of a list in our dummy question, failed to do so (*n* = 1,955); those who chose every item in a list of 7 substances (alcohol, sleeping medications, opioids, sniffing paint thinner, legal-high drugs, marijuana, and cocaine/heroin) (*n* = 476); or those who chose every item in a list of 16 diseases (*n* = 187). Following these exclusions, the data of 25,482 respondents (age range, 15–79 y; 49.7% were men) were included in the analysis.

### Dental Pain within the Month

Dental pain was assessed by the following single question: “Have you had dental pain within the last month?” and the respondents chose their answer (yes or no). A similar question has been used in previous studies (Kuhnen et al. 2009; Tsuchiya et al. 2019).

### Exposure Variables

The COVID-19 pandemic impact on socioeconomic conditions was assessed regarding household income change, work reduction, and job loss. Household income change was evaluated with the following question: “Having your previous household income as 100, how has your current household income changed? For example, answer 50 if it has reduced by half or answer 200 if it has doubled.” Respondents either provided a number or chose the option “do not know.” The variable was categorized into 4 groups: reduced, did not change, increased, and do not know. Information on work reduction and job loss was gathered by asking respondents who were employed (including self-employment) at the time of the survey using the following multiple-choice question: “Since April 2020, have you had any of the following experiences due to the COVID-19 pandemic? 1) planned work reduced or canceled; 2) suspension or temporary lay off from work; 3) job lost or contract terminated.” Respondents who answered yes to either 1) or 2) were categorized as “work reduction,” while those who answered yes to 3) were categorized as “job loss.” Those who were not employed at the time of the survey were categorized as “not employed at the time of the survey.”

### Mediator Variables

The K6 scale was used to assess psychological distress. This consists of 6 questions with a score ranging from 0 to 4 and measures nonspecific psychological distress during the past 30 d. The total score ranges from 0 to 24, and a higher score indicates more severe psychological distress. This variable was dichotomized to indicate having severe distress (K6 score: ≥13) (Kessler et al. 2003).

Oral health–related behaviors regarding toothbrushing behaviors, between-meals eating behaviors, and postponing dental visits were assessed. The following multiple-choice question was used to assess toothbrushing and between-meals eating behaviors: “Compared to January 2020 or before, how have your behaviors changed regarding the following? 1) frequency or time of toothbrushing; 2) frequency or amount of eating between meals” with possible responses: “increased,” “did not change,” and “reduced.” These variables were dichotomized to indicate unhealthy behaviors, that is, “reduced” for toothbrushing frequency/time and “increased” for between-meals eating. Postponing dental visits was assessed using the following question: “Did you postpone dental visits between April and May 2020?” and those who answered “yes” were categorized as having experience of postponing dental visits.

### Confounding Variables

Potential confounders—namely, residential region, age, sex, educational attainment, household income in 2019, and having dental checkups within the year—were also assessed in the questionnaire. Details of the categorization of variables, except for residential region, are shown in Table 1.

**Table 1.** Demographic Characteristics of Study Participants (*n* = 25,482).

	Dental Pain within the Month, *n* (%)			
Characteristic	Total (*N* = 25,482)	No (*n* = 22,989; 90.2%)	Yes (*n* = 2,493; 9.8%)	*P* Value^a^
Age, y				<0.001
15–19	1,214 (4.8)	1,066 (87.8)	148 (12.2)	
20–29	3,211 (12.6)	2,864 (89.2)	347 (10.8)	
30–39	3,767 (14.8)	3,434 (91.2)	333 (8.8)	
40–49	4,894 (19.2)	4,371 (89.3)	523 (10.7)	
50–59	4,256 (16.7)	3,835 (90.1)	421 (9.9)	
60–69	4,243 (16.7)	3,889 (91.7)	354 (8.3)	
70–79	3,897 (15.3)	3,530 (90.6)	367 (9.4)	
Sex				0.075
Men	12,673 (49.7)	11,391 (89.9)	1,282 (10.1)	
Women	12,809 (50.3)	11,598 (90.5)	1,211 (9.5)	
Educational attainment				<0.001
College graduate or higher	12,172 (47.8)	11,064 (90.9)	1,108 (9.1)	
Some college	5,387 (21.1)	4,894 (90.8)	493 (9.2)	
High school or lower	7,861 (30.8)	6,974 (88.7)	887 (11.3)	
Other	62 (0.2)	57 (91.9)	5 (8.1)	
Household income in 2019 (thousand JPY)				<0.001
≥6,000	7,700 (30.2)	6,948 (90.2)	752 (9.8)	
3,000–5,999	7,810 (30.6)	7,010 (89.8)	800 (10.2)	
0–2,999	4,698 (18.4)	4,182 (89.0)	516 (11.0)	
Do not want to answer	2,560 (10.0)	2,369 (92.5)	191 (7.5)	
Do not know	2,714 (10.7)	2,480 (91.4)	234 (8.6)	
Dental checkup within the year				<0.001
No	13,538 (53.1)	12,400 (91.6)	1,138 (8.4)	
Yes	11,944 (46.9)	10,589 (88.7)	1,355 (11.3)	
Household income change				<0.001
Reduced	6,389 (25.1)	5,565 (87.1)	824 (12.9)	
Did not change	11,441 (44.9)	10,477 (91.6)	964 (8.4)	
Increased	755 (3.0)	665 (88.1)	90 (11.9)	
Do not know	6,897 (27.1)	6,282 (91.1)	615 (8.9)	
Work reduction				<0.001
No	10,484 (41.1)	9,580 (91.4)	904 (8.6)	
Yes	4,970 (19.5)	4,264 (85.8)	706 (14.2)	
Not employed at the time of the survey	10,028 (39.4)	9,145 (91.2)	883 (8.8)	
Job loss				<0.001
No	15,176 (59.6)	13,638 (89.9)	1,538 (10.1)	
Yes	278 (1.1)	206 (74.1)	72 (25.9)	
Not employed at the time of survey	10,028 (39.4)	9,145 (91.2)	883 (8.8)	
Severe psychological distress				<0.001
No (K6 score: 0–12)	23,374 (91.7)	21,324 (91.2)	2,050 (8.8)	
Yes (K6 score: 13–24)	2,108 (8.3)	1,665 (79.0)	443 (21.0)	
Postponing dental visits between April and May				<0.001
No	21,951 (86.1)	20,102 (91.6)	1,849 (8.4)	
Yes	3,531 (13.9)	2,887 (81.8)	644 (18.2)	
Toothbrushing frequency/time				0.027
Increased/did not change	24,724 (97.0)	22,323 (90.3)	2,401 (9.7)	
Reduced	758 (3.0)	666 (87.9)	92 (12.1)	
Between-meals eating				<0.001
Reduced/did not change	18,532 (72.7)	16,923 (91.3)	1,609 (8.7)	
Increased	6,950 (27.3)	6,066 (87.3)	884 (12.7)	

COVID-19, coronavirus disease 2019; JPY, Japanese yen.

a *P* value from χ^2^ test.

### Statistical Analysis

**Figure 1.**
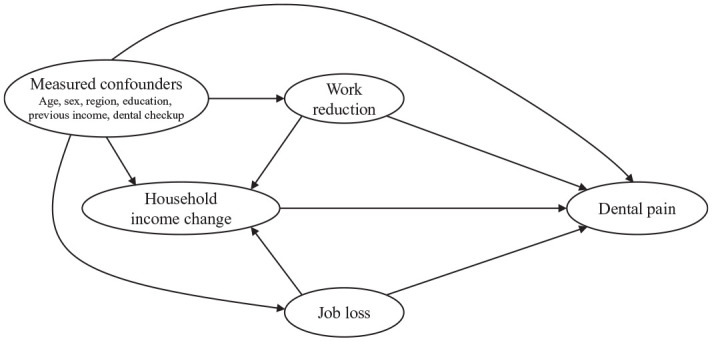
Directed acyclic graph for logistic regression analysis.

Multivariable logistic regression analysis was applied to evaluate the association between socioeconomic conditions worsened by the COVID-19 pandemic and dental pain. The directed acyclic graph (DAG) for the logistic regression analysis is shown in Figure 1. In models 1 to 3, household income change, work reduction, and job loss were separately included, adjusting for age, sex, residential region, educational attainment, household income in 2019, and dental checkup within the year. In model 4, the variables of worsened socioeconomic conditions were simultaneously included to evaluate the independent associations with dental pain.

Causal mediation analysis was employed to evaluate the extent to which psychological distress, postponing dental visits, reduced toothbrushing frequency/time, and increased frequency/amount of between-meals eating mediate the association of household income reduction with dental pain. People who reported income increment or answered “do not know” about income change were excluded from the mediation analysis. Thus, the income variable in the mediation analysis had 2 categories: reduced or did not change. Those who reported “other” for their educational attainment were excluded from the mediation analysis because of the convergence problem.

**Figure 2.**
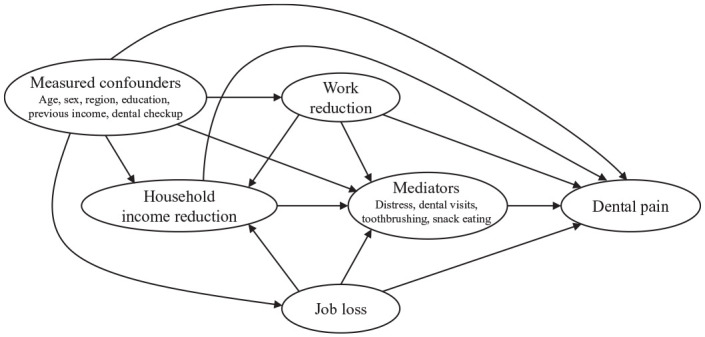
Directed acyclic graph for mediation analysis; each mediator was separately included in the mediation model.

The DAG for the mediation analysis is shown in Figure 2. Under the assumptions 1) no unmeasured exposure-outcome confounding, 2) no unmeasured mediator-outcome confounding, 3) no unmeasured exposure-mediator confounding, and 4) no exposure-induced mediator-outcome confounding, the total effect (TE) of household income reduction on dental pain is decomposed into the path through the mediator (i.e., natural indirect effect [NIE]) and the path not through the mediator (i.e., natural direct effect [NDE]) (VanderWeele 2015). The mediators were separately included in the model to evaluate NIE for each. The proportion mediated was estimated on a risk difference scale. The variables for age, sex, residential region, work reduction, job loss, educational attainment, household income in 2019, and dental checkup within the year were adjusted as covariates.

We focused on household income reduction as the exposure of interest because it is relevant to people, including nonemployees. If we consider work reduction or job loss as the exposure, assumption 4) is violated because household income reduction works as an exposure-induced mediator-outcome confounder (Fig. 2). However, as an auxiliary analysis, we used working respondents’ data and performed a mediation analysis with work reduction or job loss as the exposure. The 95% confidence intervals (CIs) were estimated by bootstrap with 1,000 replications. We used Stata MP version 16.1 (StataCorp) for all analyses. The command “paramed” was used for the causal mediation analysis. We followed Strengthening the Reporting of Observational Studies in Epidemiology (STROBE) guidelines.

## Results

Table 1 describes the demographic characteristics of the respondents. The prevalence of dental pain within a month was 9.8%. Among the 3 items indicating the impact of the COVID-19 pandemic on socioeconomic conditions, household income reduction was the most frequently reported (25.1%), followed by work reduction (19.5%). Job loss was reported by 1.1% of the respondents. All 3 items were significantly associated with dental pain. Reduced toothbrushing frequency/time, increased between-meals eating frequency/amount, postponing dental visit, and psychological distress were significantly associated with dental pain. Lower educational attainment and lower household income were also significantly associated with dental pain.

Table 2 reports the association of socioeconomic conditions worsened by the COVID-19 pandemic with dental pain. As models 1 to 3 show, dental pain was significantly associated with household income reduction (odds ratio [OR], 1.58; 95% CI, 1.43–1.75), work reduction (OR, 1.76; 95% CI, 1.59–1.96), and job loss (OR, 2.90; 95% CI, 2.20–3.83) after adjusting for age, sex, residential region, educational attainment, household income in 2019, and dental checkup within the year. Income increment was also associated with dental pain (OR, 1.41; 95% CI, 1.12–1.78). People from a lower socioeconomic background (i.e., lower household income in 2019 and lower educational attainment) were more likely to have dental pain. Household income reduction, work reduction, and job loss were independently associated with dental pain (model 4). More specifically, the DAG (Fig. 1) suggested that household income reduction was associated with dental pain after adjusting for work reduction and job loss (OR, 1.42; 95% CI, 1.28–1.57); work reduction and job loss were associated with dental pain through the pathway other than household income change (OR, 1.58; 95% CI, 1.41–1.76; and OR, 2.17; 95% CI, 1.64–2.88, respectively).

**Table 2.** Association between Worsened Socioeconomic Conditions Due to the COVID-19 Pandemic and Dental Pain (*n* = 25,482).

Characteristic	Model 1, OR (95% CI)	Model 2, OR (95% CI)	Model 3, OR (95% CI)	Model 4, OR (95% CI)
Household income change				
Reduced	1.58 (1.43–1.75)			1.42 (1.28–1.57)
Did not change	Reference			Reference
Increased	1.41 (1.12–1.78)			1.31 (1.04–1.66)
Do not know	1.10 (0.98–1.23)			1.10 (0.98–1.24)
Work reduction				
No		Reference		Reference
Yes		1.76 (1.59–1.96)		1.58 (1.41–1.76)
Not employed at the time of the survey		0.97 (0.87–1.09)		0.98 (0.88–1.10)
Job loss				
No			Reference	Reference
Yes			2.90 (2.20–3.83)	2.17 (1.64–2.88)
Not employed at the time of the survey			0.82 (0.74–0.91)	
Educational attainment				
College graduate or higher	Reference	Reference	Reference	Reference
Some college	1.05 (0.94–1.19)	1.08 (0.96–1.22)	1.05 (0.94–1.19)	1.07 (0.95–1.20)
High school or lower	1.33 (1.20–1.47)	1.37 (1.24–1.52)	1.33 (1.21–1.47)	1.36 (1.23–1.50)
Other	0.85 (0.34–2.14)	0.85 (0.34–2.14)	0.83 (0.33–2.09)	0.83 (0.33–2.09)
Household income in 2019 (thousand JPY)				
≥6,000	Reference	Reference	Reference	Reference
3,000–5,999	1.07 (0.96–1.19)	1.10 (0.99–1.23)	1.09 (0.98–1.21)	1.08 (0.97–1.21)
0–2,999	1.15 (1.02–1.31)	1.23 (1.09–1.40)	1.20 (1.06–1.36)	1.19 (1.05–1.35)
Do not want to answer	0.80 (0.68–0.96)	0.83 (0.70–0.98)	0.80 (0.68–0.95)	0.83 (0.70–0.99)
Do not know	0.90 (0.76–1.07)	0.93 (0.79–1.09)	0.90 (0.77–1.06)	0.94 (0.79–1.11)

All models were adjusted for listed variables and age, sex, residential region (Hokkaido/Tohoku, Kanto, Chubu, Kinki, Chugoku/Shikoku, Kyushu/Okinawa), and dental checkup within the year.

COVID-19, coronavirus disease 2019; JPY, Japanese yen; OR, odds ratio.

The results from the mediation analysis are reported in Table 3. The association of household income reduction with dental pain was mediated by psychological distress by 21.3% (95% CI, 14.0%–31.6%), postponing dental visits by 12.4% (95% CI, 7.2%–19.6%), toothbrushing behavior by 1.5% (95% CI, −0.01% to 4.5%), and between-meals eating behavior by 9.3% (95% CI, 5.4%–15.2%), respectively. NDE of household income reduction remained significant in each mediation model. Our auxiliary analysis showed that postponing dental visits mediated the association related to work reduction by 17.3% (95% CI, 10.9%–25.4%) (Appendix Table 1). The association related to job loss was mediated by psychological distress by 34.3% (95% CI, 8.1%–78.1%) and postponing dental visits by 21.3% (95% CI, 5.7%–48.7%) (Appendix Table 2).

**Table 3.** Effect Decomposition of Total Effect of Income Reduction Due to COVID-19 on Dental Pain (*n* = 17,797).

Mediator^a^	TE, OR (95% CI)^b^	NDE, OR (95% CI)^b^	NIE, OR (95% CI)^b^	PM (95% CI)^b,c^
Psychological distress: yes	1.42 (1.27–1.57)	1.33 (1.20–1.48)	1.07 (1.05–1.09)	21.3 (14.0–31.6)
Postponing dental visits: yes	1.43 (1.29–1.59)	1.38 (1.24–1.53)	1.04 (1.02–1.06)	12.4 (7.2–19.6)
Toothbrushing frequency/time: reduced	1.41 (1.27–1.58)	1.41 (1.27–1.57)	1.005 (1.000–1.012)	1.5 (–0.01 to 4.5)
Between-meals eating: increased	1.42 (1.28–1.58)	1.38 (1.24–1.54)	1.03 (1.02–1.04)	9.3 (5.4–15.2)

All models were adjusted for age, sex, residential region (Hokkaido/Tohoku, Kanto, Chubu, Kinki, Chugoku/Shikoku, Kyushu/Okinawa), work reduction, job loss, educational attainment, household income in 2019, and dental checkup within the year. People who reported income increment were excluded. People who answered “other” for educational attainment were excluded for convergence in each bootstrap replication.

NDE, natural direct effect; NIE, natural indirect effect; OR, odds ratio; PM, proportion mediated; TE, total effect.

a Each mediator was separately included.

b Estimated by bootstrap with 1,000 replications.

c Proportion mediated on a risk difference scale.

## Discussion

The present study found that socioeconomic conditions worsened by the COVID-19 pandemic were associated with dental pain among the Japanese population. The association related to household income reduction was mediated by psychological distress, postponing dental visits, toothbrushing behavior, and between-meals eating behavior by 21.3%, 12.4%, 1.5%, and 9.3%, respectively.

To the best of our knowledge, the present study is the first to report the economic impact of the COVID-19 pandemic on dental health outcomes. Although previous studies have reported the characteristics of patients who visited emergency dental care during the national lockdown (Bai et al. 2020; Samuel et al. 2021) and infection control in dental clinics to reduce person-to-person transmission (Zemouri et al. 2020), the impact of COVID-19 on dental health has not been evaluated. A study from Chennai, India, reported that dental pain, fear of COVID-19, and psychological distress were associated with low oral health–related quality of life (OHRQOL) (Samuel et al. 2021); they failed to compare people with and without dental symptoms as the study participants were patients who visited a dental institute for emergency care. Although few studies are directly comparable to ours, the findings are supported by a previous study conducted following another crisis, the devastating 2011 earthquake in Japan, showing that survivors who had lost their properties were more likely to report dental pain (Tsuchiya et al. 2019).

Household income reduction was associated with dental pain via psychological distress. This is in line with a previous study reporting that higher anxiety and depression under the COVID-19 pandemic were more prevalent among those who lost their income (Hertz-Palmor et al. 2020). A lowered immune system due to psychological distress increases periodontal inflammation (Peruzzo et al. 2007) and might lead to dental pain. Although we could not evaluate the sequence among mediators in this cross-sectional study, people in economic hardship may develop unhealthy eating behaviors to cope with the stress of their situation (Spinosa et al. 2019), which may lead to dental caries and dental pain. If we were able to consider the mediators simultaneously, NIEs of household income reduction through toothbrushing and eating behaviors might reduce. The homebound lifestyle during lockdown would also have contributed to unhealthy eating behavior (Carroll et al. 2020).

Unexpectedly, household income increment was also associated with dental pain. Although we could not evaluate this further because of the small number of people with an income increment, the result might reflect a demand for continued working in some occupations that increased due to the pandemic. For example, people working at information technology companies may be required to work harder to develop teleworking infrastructure, leading to income increment as well as psychological distress. Further research is needed to evaluate the reason for the association of income increment and dental pain.

The association between lack of dental service use and dental pain has been previously reported (Kuhnen et al. 2009). Our findings showed that postponing dental visits mediated the association of worsened socioeconomic conditions and dental pain. The recommendations/suggestions from dental organizations for postponement of nonacute dental visits (COVID-19 Dental Services Evidence Review Working Group 2020) might have influenced dental health care seeking. Although the closure was not mandated in Japan, and few dental clinics closed (Tada et al. 2020), about 50% of patients canceled their planned dental visits (Koyama and Takeuchi 2020).

Our logistic regression analysis showed independent associations of work reduction and job loss with dental pain. During the COVID-19 lockdown, people in lower occupational classes were more likely to have economic hardship and depressive symptoms (Witteveen and Velthorst 2020). The expectation of job loss as a result of the pandemic was associated with depressive symptoms among young adults (Ganson et al. 2021). Although it should be interpreted with caution, our auxiliary analysis for working respondents showed that the association related to job loss was mediated by psychological distress by 34.3%. Economic hardship and anxiety for the future could have increased psychological distress and might lead to dental pain. On the other hand, psychological distress did not largely mediate the association related to work reduction. Postponing dental visits was suggested as the pathway linking work reduction with dental pain. However, under our analytic framework, it was difficult to distinguish the mechanisms related to each measure of the worsened socioeconomic condition.

There is concern that existing oral health inequalities would become worse during the pandemic (Watt 2020). The present study suggested that the COVID-19 pandemic has already affected dental pain. Policies protecting income and jobs would contribute to reducing dental health problems. Dental diseases are prevalent across all age groups (GBD 2017 Oral Disorders Collaborators et al. 2020), and it should be evaluated whether dental caries and periodontal diseases increase long term. Dental services need to be provided under infection control. Furthermore, preventive approaches generally reduce the need for invasive, aerosol-generating procedures. Upstream dental health approaches will be required in a society that has experienced a historical pandemic (Watt 2020).

The present study has limitations. First, given the cross-sectional study design, the temporality between variables is not guaranteed. However, it would be less likely that dental pain within the month worsened socioeconomic conditions due to the COVID-19 pandemic. Thus, the association between worsened socioeconomic conditions and dental pain would be robust to reverse causation. On the other hand, the NIE for each mediator might be overestimated. Dental pain could induce psychological distress, or dental pain might change oral health–related behaviors. Second, we could not evaluate the mediating effects simultaneously because the method is under development and has not been implemented in practice (VanderWeele 2015). It could be speculated that the mediators are correlated with each other, and thus, each NIE could reduce if they were included simultaneously; however, the direction of bias depends on the relationship with other variables, including unmeasured confounders.

Third, we used self-reported information, and some of our measurements have not been validated. The survey was developed to cover broad topics rather than focusing on dental health–related issues, which led to some difficulties. For example, we could not assess whether those who had postponed dental visits in April or May still had the same problem at the time of the survey (i.e., August to September). The question on work reduction and job loss was applied to those who were working at the time of the survey; therefore, we could not capture the experience of work reduction and job loss among people who had lost their job due to the pandemic and were unemployed at the time of the survey. Thus, the association of work reduction and job loss with dental pain would be underestimated.

Fourth, although participants were recruited to represent the Japanese population in terms of age, sex, and residential prefecture, the respondents of a web-based survey might not fully represent the Japanese population. To evaluate the generalizability, we compared the respondents’ demographic characteristics with national statistics (Appendix Table 3). The distribution of age, sex, and residential region and proportion of people employed at the time of the survey were similar to the relevant national surveys. Study respondents’ educational attainment and household income leaned toward high. Given that the COVID-19 pandemic has severely affected vulnerable populations, our results might underestimate the pandemic’s impact on dental pain.

In conclusion, the present study found that people whose socioeconomic conditions worsened due to the COVID-19 pandemic were more likely to report dental pain. Policies that mitigate the negative impact of the pandemic would prevent worsening dental diseases.

## Author Contributions

Y. Matsuyama, contributed to conception, data analysis, and interpretation, drafted the manuscript; J. Aida, S. Koyama, K. Takeuchi, contributed to conception and data interpretation, critically revised the manuscript; T. Tabuchi, contributed to conception, design, data acquisition, analysis, and interpretation, critically revised the manuscript. All authors gave final approval and agree to be accountable for all aspects of the work.

## Supplemental Material

sj-pdf-1-jdr-10.1177_00220345211005782 – Supplemental material for Dental Pain and Worsened Socioeconomic Conditions Due to the COVID-19 Pandemic Click here for additional data file. Supplemental material, sj-pdf-1-jdr-10.1177_00220345211005782 for Dental Pain and Worsened Socioeconomic Conditions Due to the COVID-19 Pandemic by Y. Matsuyama, J. Aida, K. Takeuchi, S. Koyama and T. Tabuchi in Journal of Dental Research
