# Supplementary material for: Dental Pain and Worsened Socioeconomic Conditions Due to the COVID-19 Pandemic
Source: J Dent Res. 2021 Apr 1;100(6):591–8. doi: 10.1177/00220345211005782 (PMC8138328; doi:10.1177/00220345211005782)
Supplement: sj-pdf-1-jdr-10.1177_00220345211005782 – Supplemental material for Dental Pain and Worsened Socioeconomic Conditions Due to the COVID-19 Pandemic [file sj-pdf-1-jdr-10.1177_00220345211005782.pdf]

Appendix Table 1. Effect decomposition of total effect of work reduction due to COVID-19 on dental pain (n = 11,403[ab])

Mediators[c]	TE	NDE	NIE	PM[d]
	OR (95% CI[e])	OR (95% CI[e])	OR (95% CI[e])	% (95% CI[e])
Psychological distress: yes	1.59 (1.39, 1.81)	1.56 (1.36, 1.78)	1.015 (1.001, 1.033)	4.1 (0.2, 8.8)
Postponing dental visits: yes	1.59 (1.39, 1.81)	1.49 (1.30, 1.71)	1.07 (1.05, 1.10)	17.3 (10.9, 25.4)
Toothbrushing frequency/time: reduced	1.58 (1.39, 1.80)	1.58 (1.39, 1.80)	0.999 (0.993, 1.000)	-0.3 (-2.3, 0.1)
Between-meals eating: increased	1.58 (1.39, 1.80)	1.57 (1.37, 1.79)	1.01 (0.99, 1.03)	2.8 (-1.5, 8.1)

Abbreviations: confidence interval, CI; odds ratio, OR; total effect, TE; natural direct effect, NDE; natural indirect effect, NIE; proportion mediated, PM

All models were adjusted for age, sex, residential region (Hokkaido/Tohoku, Kanto, Chubu, Kinki, Chugoku/Shikoku, Kyushu/Okinawa), household income reduction, job loss, educational attainment, household income in 2019, and dental checkup within the year

a People who reported income increment and those who were unemployed at the time of the survey were excluded

b People answered “other” for educational attainment were excluded for convergence in each bootstrap replication

c Each mediator was separately included

d Proportion mediated on a risk difference scale

e Estimated by bootstrap with 1,000 replications

Appendix Table 2. Effect decomposition of total effect of job loss due to COVID-19 on dental pain (n = 11,403[ab])

Mediators[c]	TE	NDE	NIE	PM[d]
	OR (95% CI[e])	OR (95% CI[e])	OR (95% CI[e])	% (95% CI[e])
Psychological distress: yes	2.10 (1.45, 2.91)	1.72 (1.10, 2.51)	1.22 (1.04, 1.55)	34.3 (8.1, 78.1)
Postponing dental visits: yes	2.24 (1.55, 3.16)	1.98 (1.34, 2.76)	1.13 (1.03, 1.36)	21.3 (5.7, 48.7)
Toothbrushing frequency/time: reduced	2.20 (1.50, 3.06)	1.98 (1.35, 2.71)	1.11 (1.01, 1.40)	18.3 (1.3, 49.1)
Between-meals eating: increased	2.18 (1.49, 2.97)	2.09 (1.44, 2.89)	1.04 (0.99, 1.17)	7.6 (-1.4, 29.2)

Abbreviations: confidence interval, CI; odds ratio, OR; total effect, TE; natural direct effect, NDE; natural indirect effect, NIE; proportion mediated, PM

All models were adjusted for age, sex, residential region (Hokkaido/Tohoku, Kanto, Chubu, Kinki, Chugoku/Shikoku, Kyushu/Okinawa), household income reduction, work reduction, educational attainment, household income in 2019, and dental checkup within the year

a People who reported income increment and those who were unemployed at the time of the survey were excluded

b People answered “other” for educational attainment were excluded for convergence in each bootstrap replication

c Each mediator was separately included

d Proportion mediated on a risk difference scale

e Estimated by bootstrap with 1,000 replications

Appendix Table 3. Comparison of demographic characteristics between the study respondents and other national surveys

	Study respondents	National surveys[a]
	n (%)	%
Age (years)		
15–19	1,214 (4.8%)	6.0%
20–29	3,211 (12.6%)	12.4%
30–39	3,767 (14.8%)	15.3%
40–49	4,894 (19.2%)	18.8%
50–59	4,256 (16.7%)	15.3%
60–69	4,243 (16.7%)	18.3%
70–79	3,897 (15.3%)	13.8%
Sex		
Men	12,673 (49.7%)	49.6%
Women	12,809 (50.3%)	50.4%
Residential region		
Hokkaido, Tohoku	2788 (10.9%)	11.1%
Kanto	9007 (35.4%)	34.7%
Chubu	4238 (16.6%)	16.7%
Kinki	4521 (17.7%)	17.8%
Chugoku, Shikoku	2140 (8.4%)	8.6%
Kyushu	2788 (10.9%)	11.0%
Educational attainment		
College graduate or more	12,172 (47.8%)	17.3%
Some college	5,387 (21.1%)	12.9%
High school or less	7,861 (30.8%)	56.8%
Other	62 (0.2%)	13.1%
Household income (thousand JPY)		
≥6,000	7,700 (30.2%)	34.2%
3,000–5,999	7,810 (30.6%)	32.4%
0–2,999	4,698 (18.4%)	33.3%
Do not want to answer	2,560 (10.0%)	-
Do not know	2,714 (10.7%)	-
Employment status		
Employed/self-employed	15,454 (60.6%)	63.3%
Unemployed	10,028 (39.4%)	36.7%

a Source: Population estimate 2016 for age, sex, and residential region; National census 2010 for educational attainment; and Comprehensive Survey of Living Conditions of People on Health and Welfare 2016 for household income and employment status.
